# Prevalence and Mortality Risk of Persistent Smoking After Myocardial Infarction in a Very-High-Risk Region of Southeastern Europe

**DOI:** 10.3390/medicina62071357

**Published:** 2026-07-14

**Authors:** Aleksandra Milovančev, Aleksandra Ilić, Tatjana Miljković, Snežana Čemerlić Maksimović, Isidora Milosavljević, Aleksandra Matić, Milovan Petrović

**Affiliations:** 1 Faculty of Medicine, University of Novi Sad, 21000 Novi Sad, Serbia; aleksandra.ilic@mf.uns.ac.rs (A.I.);; 2 Institute for Cardiovascular Diseases of Vojvodina, 21204 Sremska Kamenica, Serbia; 3 Faculty of Sport and Physical Education, University of Novi Sad, 21000 Novi Sad, Serbia; 4 Faculty of Medicine, Semmelweis University, 1085 Budapest, Hungary

**Keywords:** myocardial infarction, smoking, all-cause mortality

## Abstract

*Background and Objectives*: Long-term real-world data regarding smoking cessation after myocardial infarction (MI) remain scarce in Southeastern Europe. Our objective was to estimate the prevalence of smoking status categories and to assess their association with long-term all-cause mortality in percutaneous coronary intervention (PCI) treated MI patients. *Materials and Methods*: We retrospectively collected data from electronic medical records of hospitalized MI patients who underwent PCI from 1 January 2016 to 1 January 2019. Smoking status and primary outcome (all-cause mortality) were assessed at baseline, during follow-up visits, and during teleconsultation until January 2024, and patients were categorized accordingly. *Results*: Never-smokers were older than persistent smokers (67.6 ± 11.4 vs. 57.3 ± 9.6 years; *p* < 0.01) and had a higher burden of hypertension and diabetes (both *p* < 0.01). At baseline, 47.9% were active smokers, 32.3% of them quit after an MI (with 77.7% being men). Over a median follow-up of 6.7 years, 220 deaths (17.3%) occurred. In a Cox regression model adjusted for age, persistent post-MI smoking significantly increased mortality risk (aHR 2.18; 95% CI 1.52–3.12; *p* < 0.001), former smokers had an aHR was 1.07 (*p* = 0.694), while quitters had 30% reduction in death risk (aHR 0.69; *p* = 0.217). Additionally, in the fully adjusted model, besides persistent smoking (aHR 2.18; *p* = 0.001), diabetes (aHR 1.69; *p* = 0.001) and age (aHR 1.09; *p* < 0.001) were independent predictors of all-cause mortality. *Conclusions*: Post-MI smoking more than doubles mortality risk, while sustained cessation reduces risk to near never-smoker levels, underscoring the need for aggressive, tailored cessation strategies in low-quit-rate regions such as Southeastern Europe.

## 1. Introduction

According to the World Health Organization, tobacco use is responsible for approximately eight million deaths annually [1]. Nearly one-half of smoking-attributable deaths are considered preventable. Smoking is a leading modifiable risk factor for atherosclerotic cardiovascular disease, promoting endothelial dysfunction, inflammation, thrombosis, and accelerated atherosclerosis, thus increasing the risk of acute myocardial infarction (AMI) and stroke [2]. Some previous studies reported a phenomenon called “smoker’s paradox,” which refers to the observation that smokers may appear to have more favorable short-term outcomes after STEMI, which was historically attributed to potential protective effects of smoking on infarct size. However, this apparent paradox is more likely explained by confounding, as smokers are often younger and have fewer baseline cardiovascular risk factors than nonsmokers [3,4]. Among patients who survive an AMI, continued tobacco use remains a major determinant of long-term prognosis. Observational evidence consistently demonstrates that smoking cessation after AMI is associated with substantially lower mortality compared with persistent smoking [5]. Hence, contemporary secondary prevention and acute coronary syndrome guidelines emphasize systematic assessment of tobacco use, structured counselling, and pharmacological support to achieve complete abstinence [6]. However, evidence on the real-world implementation and effectiveness of these recommendations remains limited, particularly in Southeastern European countries where cardiovascular risk is very high.

Although numerous studies link smoking to higher mortality, direct comparisons remain limited by heterogeneous study designs and varied exposure assessments. Key discrepancies include whether smoking status was assessed solely at baseline or longitudinally during follow-up, the specific definitions applied to cessation and persistence, and the overall duration of follow-up. For instance, in a benchmark meta-analysis including 9527 patients [7], smoking status was commonly assessed only at baseline, or, in other reports, reassessed at 6 weeks [8] or at 1, 6, and 12 months after AMI, and then correlated with subsequent outcomes [9]. Specifically, a substantial proportion of patients who stop smoking immediately after revascularization and percutaneous coronary intervention (PCI) relapse within 3–6 months; without repeated assessment, studies may misclassify relapse as sustained cessation and consequently underestimate the true benefit of quitting. To date, data on the long-term prevalence of persistent smoking after AMI, despite guideline-recommended cessation interventions, and its association with outcomes in this part of Southeastern Europe remain scarce. The study aimed to estimate the prevalence of post-AMI smoking status categories and to assess their association with long-term all-cause mortality in PCI-treated patients.

## 2. Materials and Methods

In this observational retrospective cohort study, we analyzed data from patients admitted to the university’s tertiary care high-volume center with a principal diagnosis of AMI between 1 January 2016 and 1 January 2019. Consecutive patients with ST-segment elevation AMI (STEMI) or non–ST-segment elevation AMI (NSTEMI) who underwent PCI were eligible. AMI was defined according to the Fourth Universal Definition of Myocardial Infarction [10]. Data were extracted from electronic medical records, including cardiovascular risk factors, comorbidities, clinical status at admission, length of stay, echocardiographic parameters, in-hospital mortality, and others. Smoking status and smoking cessation were the exposure variables. Prespecified potential confounders were age, sex, hypertension, diabetes mellitus, dyslipidemia, and discharge medications (aspirin, P2Y12 inhibitors, and statins). Specifically, we tracked antiplatelet and statin therapies prescribed at discharge. All acetylsalicylic acid medicaments were classified as Aspirin and clopidogrel, ticagrelor, and prasugrel as P2Y12 inhibitors, which were extracted as categorical exposure variables and included in the multivariable models to adjust for potential confounding.

Participants were followed until the occurrence of the primary endpoint, all-cause mortality, or, if event-free, until 12 January 2024. The date of death was ascertained from hospital records or, when unavailable, by telephone interview with a family member. For patients without a recorded event in the electronic database, follow-up information was obtained by telephone interview. Smoking status was obtained from the electronic record at the index hospitalization and reassessed at 1, 6 and 12 months after the index hospitalization, and reassessed at study completion (12 January 2024). Scheduled follow-up assessments were performed in outpatient visits; when visits were missed, data were collected by telephone interview with patients or family. Patients were categorized into four groups: never-smokers (no history of regular cigarette smoking), former smokers (cessation ≥6 months before the index hospitalization), quitters (permanent cessation within 1 month after the index hospitalization), and persistent smokers (smoked both before and after the index hospitalization). Patients who initiated smoking during follow-up or who quit beyond the predefined post-index window were excluded. Patients with insufficient information on smoking status were also excluded. Outcomes were analyzed according to smoking category. Selection flow: A total of 4210 patients with AMI treated with PCI and documented baseline smoking status were screened. Patients were sequentially excluded using non-overlapping criteria: 50 patients who initiated smoking after AMI, 140 with uncertain smoking status, 12 with an unknown exact date of death, 1749 with incomplete clinical data, and 990 who were lost to follow-up, yielding a final study cohort of 1269 patients. One investigator performed a final data quality check for missing or inconsistent entries and values outside expected ranges. The study was conducted in accordance with the Declaration of Helsinki and was approved by the Institutional Ethics Committee of the participating institution under number 2865-1/9.

### Statistical Analysis

Continuous variables are presented as mean ± standard deviation (SD) or median (25th–75th percentile), as appropriate. Normality was assessed using the Kolmogorov–Smirnov test. Categorical variables are presented as counts and percentages. Differences across the four smoking groups were evaluated using one-way analysis of variance (ANOVA) for continuous variables and the chi-square test for categorical variables. Survival curves were estimated using the Kaplan–Meier method and compared across smoking status groups using the log-rank test. Multivariable Cox proportional hazards regression was performed to estimate adjusted hazard ratios (aHR) with 95% confidence intervals (CI), adjusting for age as a continuous covariate. In our fully adjusted multivariable models, the association between smoking status and outcomes was controlled for prespecified confounders (age, sex, hypertension, diabetes mellitus, dyslipidemia, and medications including aspirin, P2Y12 inhibitors, and statins). The proportional hazards assumption was verified using Schoenfeld residuals, while the linearity of the age effect was assessed and confirmed using restricted cubic splines. A two-sided *p*-value < 0.05 was considered statistically significant, and analyses were performed using Stata 18 (StataCorp, College Station, TX, USA).

## 3. Results

In total, 1269 patients hospitalized with AMI who underwent PCI were included in the final analysis. The mean follow-up duration was 6.2 ± 1.7 years, with a median follow-up of 6.7 years, [IQR] 5.8–7.2 years, and the total observation period extended up to 7.9 years. The mean age at the index hospitalization was 62.2 ± 11.4 years overall and differed significantly by smoking status (*p* < 0.01), with persistent smokers being the youngest (57.3 ± 9.6 years) and never-smokers the oldest (67.6 ± 11.4 years). The study population was predominantly male (64.6%).

### 3.1. Risk Factors and Comorbidities

Baseline patient characteristics, stratified by smoking categories, are summarized in Table 1. The cohort of patients with AMI exhibited a high burden of cardiovascular risk factors. Arterial hypertension presented in 61% of the cohort, with significant differences across groups; the highest prevalence was observed among never-smokers (67.6%) and the lowest among persistent smokers (57.1%; *p* = 0.01). Smoking was the second most common risk factor, with a baseline prevalence of 47.9%. A positive family history of cardiovascular disease was reported by 26.7% of the total cohort, while known dyslipidemia was present in 23%. Diabetes mellitus was observed in 19.2% of all patients and was significantly more prevalent among never-smokers (24.0%) compared to former smokers (21.2%), while persistent smokers and quitters exhibited identical lower prevalences (15.2% for both; *p* < 0.01).

### 3.2. Smoking Status and Smoking History

At the time of index hospitalization, 47.9% of participants were active smokers, 20.8% were former smokers, and 31.2% were never smokers. The median smoking duration was 38 years ([IQR] 30–45), and the median age at smoking initiation was 18 years ([IQR] 16–22). Pronounced sex-based differences in smoking habits were observed across the cohorts (*p* = 0.01). While the never-smoker group was predominantly composed of women (52.7%), men constituted the majority in all other categories, representing 73.9% of former smokers, 77.7% of those who quit after the MI, and 69.0% of persistent smokers. Following the index AMI, the successful post-discharge cessation rate among baseline smokers was 32.3% (quitters); of these, 77.7% were men and 22.3% were women (*p* < 0.01).

### 3.3. Clinical Characteristics

Table 2 summarises clinical characteristics at index hospitalization stratified by smoking status. Killip class differed significantly across groups (*p* < 0.01): former smokers were more likely to present with advanced acute heart failure (Killip III: 8.2%; Killip IV: 4.9%), whereas Killip I predominated in the remaining groups. The total length of hospital stay demonstrated significant inter-group differences (*p* < 0.01); never-smokers (6.8 ± 4.7 days) and former smokers (6.8 ± 5.5 days) required similar hospitalization time, whereas persistent smokers required the shortest stay (5.9 ± 3.4 days). Mean systolic blood pressure differed across groups (*p* < 0.01), with the highest values among never-smokers (141.0 ± 27.2 mmHg), followed by former smokers (139.7 ± 27.0 mmHg), quitters (138.9 ± 25.0 mmHg), and persistent smokers (137.1 ± 23.0 mmHg). Diastolic blood pressure followed a similar trend, but without a statistically significant difference (*p* = 0.2). Heart rate also differed significantly (*p* < 0.01), with higher values in never-smokers (82.9 ± 20.9 bpm) and former smokers (82.3 ± 21.4 bpm), and comparable values in quitters and persistent smokers (80.7 ± 18.7 and 80.7 ± 18.8 bpm, respectively). Guideline-directed medical therapy at discharge demonstrated high overall adherence across the entire cohort. However, statistically significant differences emerged in the prescription of aspirin (*p* = 0.02) and P2Y12 inhibitors (*p* = 0.01), with the highest uptake observed in the quitter cohort and the lowest in former smokers.

### 3.4. Survival Analysis

**Figure 1:**
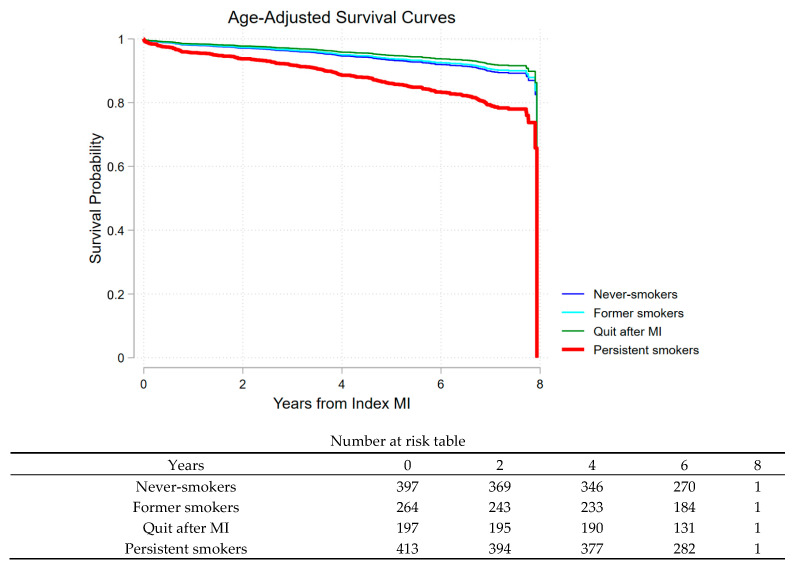
Age-adjusted survival curves. Survival estimates were derived from a multivariable Cox model, adjusted for age at its mean. Persistent smokers (red line) show significantly lower survival compared to never-smokers (blue line). Former smokers (cyan line) and quitters (green line) exhibit survival trajectories comparable to those of never-smokers. The number at risk at each 2-year interval is provided in the risk table above. The decline in curves at the end of the follow-up period (Year 8) reflects the censoring of the remaining study participants.

Over a median follow-up of 6.7 years ([IQR] 5.8–7.2 years), a total of 220 deaths (17.3%) occurred. Kaplan–Meier survival curves demonstrated significant differences in unadjusted overall survival across the four smoking cohorts (log-rank, *p* = 0.0004). During the follow-up period, crude all-cause mortality rates were 17.4% among persistent smokers, 7.6% among quitters, 17.8% among former smokers, and 21.8% among never-smokers. In the unadjusted analysis, a ‘smoking paradox’ was initially observed, where persistent smokers appeared to have a lower mortality hazard compared to never-smokers. However, after adjusting for age (Table 3), this paradox was resolved, revealing current smoking as an independent predictor of mortality (aHR 2.00; 95% CI 1.40–2.82; *p* < 0.001). The adjusted curves demonstrate (Figure 1) that persistent smokers (red line) have the poorest long-term prognosis. In contrast, former smokers (green line) and never smokers (blue line) maintained significantly higher survival probabilities. Regarding other smoking categories, former-smokers (Group 1) showed a non-significant 7% increase in mortality risk (aHR = 1.07; 95% CI 0.75–1.54; *p* = 0.694). Conversely, those who had quit smoking (Group 2) demonstrated a nearly 30% reduction in the hazard of death relative to never-smokers, although this did not reach statistical significance in the adjusted model (aHR = 0.69; 95% CI 0.39–1.24; *p* = 0.205). Age was significantly associated with mortality, with the risk of death increasing by 9.4% for each additional year of life (aHR = 1.09; 95% CI 1.08–1.11; *p* < 0.001). The proportional hazards assumption was verified using Schoenfeld residuals. The global test confirmed the validity of the model (*p* = 0.073) and specifically, the hazard for current smokers remained constant over the observation period (*p* = 0.7280 for the smoking status–time interaction. The linearity of the age effect was confirmed using restricted cubic splines, which did not significantly improve model fit over a linear term (*p* = 0.808).

In the fully adjusted multivariable Cox regression model encompassing all baseline covariates and antiplatelet discharge medications (Table 4), current smoking remained associated with more than a two-fold increase in mortality risk (aHR 2.18; 95% CI 1.52–3.12; *p* < 0.001). Among other covariates, diabetes (aHR 1.69; 95% CI 1.22–2.32; *p* = 0.001) and age (aHR 1.09; 95% CI 1.07–1.11; *p* < 0.001) were independent predictors of all-cause mortality. In contrast, guideline-directed antiplatelet therapies at discharge were associated with reduced mortality; the hazard of death was reduced by 77% with Aspirin (aHR 0.23; 95% CI 0.12–0.43; *p* < 0.001) and by 59% with P2Y12 inhibitors (aHR 0.41; 95% CI 0.27–0.60; *p* < 0.001). Baseline sex, hypertension, dyslipidemia, and discharge statin utilization in this model did not exhibit independent prognostic associations with all-cause mortality (all *p* > 0.05).

Impact of smoking cessation on mortality and effect modification across clinical strata. In the multivariable logistic regression model, smoking cessation was identified as an independent predictor of long-term survival. Patients who successfully quit smoking post-myocardial infarction exhibited a 50% reduction in the odds of all-cause mortality compared to persistent smokers (OR 0.50; 95% CI 0.27–0.92; (*p* = 0.025), after adjusting for major clinical covariates. Other independent predictors of long-term mortality included advanced age (OR 1.09; 95% CI 1.07–1.11; *p* < 0.001) and lower left ventricular ejection fraction (OR 0.95; 95% CI 0.93–0.96; *p* < 0.001). To evaluate potential effect modification, formal multiplicative interaction testing was performed across key clinical strata. The protective impact of smoking cessation on long-term mortality did not significantly differ according to baseline diabetes status, biological sex, or left ventricular ejection fraction (all interaction terms *p* > 0.05).

## 4. Discussion

This study analyzed a real-world cohort of 1269 revascularized post-AMI patients followed for up to 7.9 years, providing key insights into regional tobacco exposure and its long-term prognostic impact.

Firstly, persistent smokers were 10 years younger and predominantly male (69%) with high baseline smoking prevalence (47.9%) and fewer traditional risk factors compared to non-smokers. Baseline analysis revealed a distinct risk profile across groups; never-smokers were significantly older and exhibited a higher prevalence of traditional comorbidities, such as hypertension and diabetes mellitus, compared to persistent smokers. Conversely, persistent smokers experienced their index myocardial infarction on average nearly a decade earlier than never-smokers (mean age 57.3 vs. 67.6 years), suggesting that tobacco use severely accelerates the onset of acute coronary events despite a lower initial burden of other risk factors. Our results are consistent with those of Redfors et al. [4], who pooled data from 2654 patients across 10 randomized trials and found that smokers were, on average, 10 years younger than nonsmokers and had fewer comorbidities.

Secondly, the cohort exhibited a substantial lifetime exposure to smoking, with a median duration of 38 years (IQR 30–45) and initiation typically occurring at a median age of 18 years (IQR 16–22). The observed smoking cessation rate of 32.2% in our cohort is notably lower than the 45–50% rates reported in several international registries and the EUROASPIRE V survey [11]. This discrepancy demonstrates the significant challenges in secondary prevention within Southeast European settings, where high tobacco dependence and limited access to structured cessation programs may hinder patients’ efforts to quit. In addition to this, our findings reveal a significant gender disparity in smoking cessation post-MI, with men being substantially more successful in quitting than women (77.7% vs. 22.3%, *p* < 0.01). This is consistent with other reports suggesting that women face unique psychological and physiological barriers to cessation [12]. In post-AMI populations in high-income countries, the outcomes may vary since abstinence rates for both men and women are comparable [13].

However, distinct factors influence the success of quitting attempts depending on gender. This emphasizes the need for targeted interventions that address the specific needs of both men and women in quitting smoking as part of secondary prevention. To mitigate this problem, we propose a structured, multi-phase smoking cessation pathway aligned with the latest European Society of Cardiology (ESC) guidelines [14]. First, validated intervention tools—such as motivational counseling and educational media—should be systematically utilized alongside advising about nicotine replacement therapy while the patient is still hospitalized. Incorporating gender-specific counseling into structured cardiac rehabilitation programs during the rehabilitation and recovery process could support cessation. Comprehensive programs must combine intensive behavioural therapy with close medical follow-up to effectively manage withdrawal symptoms, anxiety, and weight gain, which serve as primary drivers of relapse in female patients. Finally, given that nicotine addiction is a chronic relapsing disease, smoking status must be treated as a vital sign and systematically updated at every outpatient follow-up visit.

And the most important of our primary findings reveals that patients who continue to smoke after an MI face a two-fold increase in the risk of death compared to never-smokers. While unadjusted data initially suggested a survival advantage for smokers, this was driven by the fact that persistent smokers were significantly younger at the time of the index event—on average, a decade younger than never-smokers (57.3 vs. 67.6 years). This study effectively dismantles the historically controversial “smoking paradox”—the flawed notion that smoking offers a protective cardiovascular benefit—a phenomenon that has been repeatedly debunked [3]. Furthermore, our results highlight the substantial benefit of tobacco cessation both prior to (former smokers) and following an AMI, achieving a survival trajectory comparable to that of never-smokers, underscoring smoking cessation as a critical determinant of long-term prognosis. It is well-established that cigarette smoking is a potent driver of cardiovascular morbidity, with confirmed detrimental impacts spanning from all-cause mortality [15] to the entire spectrum of coronary artery disease, including myocardial infarction with nonobstructive coronary arteries [16]. Beyond accelerating atherogenesis, tobacco induces endothelial dysfunction, oxidative stress, and hypercoagulability, which critically elevate risks of stent thrombosis and recurrent myocardial infarction [7]. In line with our findings, this prognostic trend is also observed in younger populations, as demonstrated by Biery et al. [17], who reported a significantly higher long-term mortality risk among persistent smokers versus quitters in a post-AMI cohort younger than 50 years.

## 5. Conclusions

In conclusion, persistent smoking following a myocardial infarction is independently associated with a more than twofold increase in long-term mortality compared to never-smokers. Smoking cessation effectively reverses this excess risk, offering a profound survival benefit that approaches the baseline trajectory of never-smokers. Particularly in Southeastern Europe, where post-discharge smoking cessation rates remain critically low despite a heavy lifetime tobacco burden, these results highlight the urgent need to transition from general advice to aggressive, tailored, and individualized smoking cessation strategies within secondary cardiovascular care.

Strengths: A major strength is the robust, long-term follow-up of up to 7.9 years in a well-characterized cohort of 1269 patients evaluated at study completion. Unlike many studies that assess smoking habits solely at the time of the index myocardial infarction, we longitudinally updated and verified patients’ smoking status throughout the follow-up period. This dynamic tracking significantly reduces misclassification bias, providing a reliable assessment of how persistent smoking versus cessation affects real-world, long-term survival.

The study utilized real-world registry data reflecting unselected, consecutive clinical practice rather than tightly controlled clinical trial populations, increasing the external validity and generalizability of our findings to everyday cardiac care.

This cohort provides much-needed longitudinal data from a Southeastern European population, a region characterized by a uniquely high tobacco burden and traditionally lower cessation rates, filling a critical gap in regional epidemiological literature.

Limitations: As a single-center study, our findings reflect the protocols and patient demographics of a specific tertiary institution and may limit generalizability to other geographic regions or healthcare tiers. The retrospective nature of this study inherently limits our ability to infer causality from the observed associations. Additionally, retrospective designs are susceptible to missing data and selection bias, which could affect the generalizability of our findings.

We lacked data on electronic cigarettes or heated tobacco products; our inability to distinguish vaping from traditional combustible tobacco may limit the specificity of risk estimates. Second, smoking status during follow-up relied on patient self-reporting, which is susceptible to social desirability or recall bias, with low potential to underestimate true smoking persistence. We acknowledge missing data on coronary anatomy, which could also independently affect outcomes, as previously reported [18]. Lastly, our study is limited by the lack of detailed data on cumulative smoking exposure quantified in pack-years, which is a recognized confounder in cardiovascular outcomes.

## Figures and Tables

**Table 1:** Baseline characteristics, risk factors, and comorbidities according to smoking status.

Variables	All (*n* = 1269)	Never-Smokers (*n* = 395)	Former Smokers (*n* = 264)	Quit After MI (*n* = 197)	Persistent Smokers (*n* = 413)	*p*
Sex (male)	64.6	47.3	73.9	77.7	69	0.01
Age, years	62.2 ± 11.4	67.6 ± 11.4	64.9 ± 10.4	57.9 ± 10.3	57.3 ± 9.6	<0.01
Family history	26.7	24.1	20.8	30.9	31	<0.01
Hypertension	61	67.6	57.9	59.9	57.1	0.01
Dyslipidaemia	23	22.8	24.2	23.3	23.2	0.9
Diabetes	19.2	24	21.2	15.2	15.2	<0.01
Previous MI	10.6	8.6	11	11.7	11.6	0.4
Previous stroke	2.8	3.3	3.4	0.5	2.9	0.2
Previous PCI	5.8	4.3	5.3	7.1	6.8	0.4
CABG	2.5	2.8	3	2.5	1.9	0.8
CKD	1.7	1.5	3	1.5	1.2	0.3
Heart failure	6.7	8.1	9.5	6.1	3.9	0.02
COPD	2.8	3.3	1.5	1.5	3.6	0.2
Atrial fibrillation	2.9	3.8	3	0.5	3.1	0.2

Legend: Values are presented as percentages (%) or mean ± standard deviation (SD). Abbreviations: MI—myocardial infarction; PCI—percutaneous coronary intervention; CABG—coronary artery bypass grafting; CKD—chronic kidney disease; COPD—chronic obstructive pulmonary disease.

**Table 2:** Clinical characteristics of patients by smoking status in index hospitalization.

Clinical Characteristics	Never-Smokers (*n* = 395)	Former Smokers (*n* = 264)	Quit After MI (*n* = 197)	Persistent Smokers (*n* = 413)	*p*
BMI (kg/m^2^)	28.0 ± 4.4	28.3 ± 4.5	27.9 ± 4.5	27.5 ± 4.5	**0.4**
Killip I	73.6	68.9	76.1	76.2	**<0.01**
Killip II	19.2	18.0	20.1	19.3	
Killip III	5.0	8.2	2.2	3.4	
Killip IV	2.2	4.9	1.6	1.0	
EF (%)	47.7 ± 9.2	48.2 ± 9.2	48.0 ± 8.4	48.8 ± 8.7	**0.3**
Length of stay, days	6.8 ± 4.7	6.8 ± 5.5	6.0 ± 4.1	5.9 ± 3.4	**<0.01**
Systolic BP (mmHg)	141.0 ± 27.2	139.7 ± 27.0	138.9 ± 25.0	137.1 ± 23.0	**<0.01**
Diastolic BP (mmHg)	82.6 ± 14.7	81.7 ± 14.4	82.3 ± 13.9	81.8 ± 13.3	**0.2**
Heart rate (bpm)	82.9 ± 20.9	82.3 ± 21.4	80.7 ± 18.7	80.7 ± 18.8	**<0.01**
Aspirin (%)	98.5	96.2	99.5	99	**0.02**
P2Y12 inhibitor (%)	90.9	92	98	94	**0.01**
Statin (%)	97	97	95	96	**0.695**

Legend: Values are presented as percentages (%) or mean ± standard deviation (SD). Abbreviations: BMI—body mass index; EF—ejection fraction; BP—blood pressure; HR—heart rate.

**Table 3:** A multivariable Cox regression analysis of time to death adjusted for age.

Variables	aHR	95% CI	*p*
Smoking status (Reference: Never smoker)			
Former smokers	1.07	0.75–1.54	0.197
Quitters	0.69	0.39–1.24	0.205
Persistent smokers	2.00	1.40–2.82	<0.001
Age (per year)	1.09	1.08–1.11	<0.001

Legend: aHR—adjusted Hazard Ratio; CI—Confidence Interval. Smoking status was modeled using never-smokers as the reference group. The model was adjusted for age as a continuous variable. Global Schoenfeld test confirmed the proportional hazards assumption (*p* = 0.073).

**Table 4:** Multivariable Cox proportional hazards analysis of predictors associated with long-term mortality.

Variable	aHR	95% CI	*p*-Value
Current smokers (vs. Never)	2.18	1.52–3.12	<0.001
Age (per year)	1.09	1.08–1.11	<0.001
Diabetes (DM)	1.69	1.22–2.32	0.001
Sex (Female vs. Male)	0.81	0.61–1.10	0.188
Hypertension	0.85	0.63–1.14	0.284
Dyslipidaemia	0.80	0.56–1.15	0.240
Aspirin	0.23	0.12–0.43	<0.001
P2Y12 inhibitor	0.41	0.27–0.60	<0.001
Statin	0.67	0.31–1.44	0.311

Legend: aHR—adjusted Hazard Ratio; CI—Confidence interval. Smoking status was modeled using never-smokers as the reference group. The multivariable model was simultaneously adjusted for age (continuous), sex, and the presence of hypertension, diabetes mellitus, and hyperlipidemia, Aspirin, P2Y12 inhibitor, and statin.

## Data Availability

Data are available based on a reasonable request from the corresponding author.

## Abbreviations

The following abbreviations are used in this manuscript:

AMI	Acute myocardial infarction
PCI	Percutaneous coronary intervention
STEMI	ST-segment elevation Acute myocardial infarction
NSTEMI	non–ST-segment elevation Acute myocardial infarction
